# Juvenile primary Sjögren’s syndrome with ranula: is ranula a clinical sign that leads to early detection of Sjögren’s syndrome?

**DOI:** 10.1007/s11282-020-00473-8

**Published:** 2020-08-16

**Authors:** Yukinori Takagi, Kunio Hashimoto, Ikuo Katayama, Sato Eida, Misa Sumi

**Affiliations:** 1 Department of Radiology and Cancer Biology, Nagasaki University Graduate School of Biomedical Sciences, 1-7-1, Sakamoto, Nagasaki, 852-8588 Japan; 2 Department of Pediatrics, Nagasaki University Graduate School of Biomedical Sciences, 1-7-1, Sakamoto, Nagasaki, 852-8588 Japan

**Keywords:** Juvenile Sjögren’s syndrome, Ranula, Magnetic resonance imaging, MR sialography, Ultrasonography

## Abstract

Juvenile primary Sjögren’s syndrome (pSS) is rare. Although recurrent parotitis is reported to be the most common symptom of juvenile pSS, the clinical symptoms and features of the syndrome are not well understood and are poorly defined. Here we report a rare case of juvenile pSS in a patient with plunging ranula. The patient had no symptoms other than swelling of the oral floor and had no symptoms of parotitis. Magnetic resonance imaging (MRI) revealed the diagnosis of plunging ranula. In addition, the findings of the bilateral parotid glands on MRI and subsequent ultrasonography (US) strongly suggested SS. On the basis of these imaging findings and laboratory data, a pediatric rheumatologist confirmed the diagnosis of juvenile pSS. The ranula may be one clinical sign of SS. However, this association remains generally unknown. Hypothesizing that SS might cause ranula development, we retrospectively investigated cases of patients with ranula who underwent MRI at our hospital. We found that many of these patients (> 20%) had characteristic findings strongly suggestive of SS. This result suggests that SS-induced changes in the sublingual glands are one cause of ranula formation. We think that ranula is a sign of early-stage SS. Therefore, patients with ranulae, whether adults or children, should undergo careful assessment of not only the sublingual glands but also the parotid and submandibular glands with MRI and/or US to investigate possible SS. This assessment may lead to early detection of SS.

## Introduction

Sjögren’s syndrome (SS) is a chronic inflammatory autoimmune disease with a strong female predominance that is generally diagnosed in middle-aged and older individuals. This disorder is characterized by lymphocytic infiltration of exocrine glands, such as the salivary and lacrimal glands, resulting in dry mouth (xerostomia) and dry eyes (keratoconjunctivitis sicca), and is divided into primary SS (pSS) with no associated other autoimmune diseases and secondary SS (sSS) with other autoimmune diseases such as rheumatoid arthritis, mixed connective tissue disease, systemic lupus erythematosus, or scleroderma.

Although pSS also is often complicated by extraglandular manifestations in the liver, lung, kidney, or nervous system, in the early stage the most common clinical symptoms are dry mouth and dry eyes.

However, even these symptoms appear after the disease has progressed to some extent; almost no symptoms are seen in the initial stage. Although this lack of early symptoms indicates that the age of onset of pSS is younger than reported, the actual age of onset has not been elucidated.

Similar to adult SS, juvenile SS is divided into primary and secondary categories depending on whether or not other autoimmune diseases are associated. Most cases of juvenile SS are sSS found on systemic examination after diagnosis of other autoimmune diseases. In contrast, juvenile pSS is rare because it is difficult to diagnose.

Although reports to date indicate that sicca symptoms are less common in juvenile pSS than in adults and that the most common clinical symptom in children is recurrent parotitis [1–4], the clinical manifestations of juvenile pSS are not yet fully understood.

Here, we present a rare case of a 12 year-old boy in whom juvenile pSS was suspected on the basis of magnetic resonance imaging (MRI) that was performed for the detailed diagnosis of a ranula.

In the present case, multiple dispersed high-intensity spots in the bilateral parotid glands were found on fat-suppressed T2-weighted MR images, punctate sialoectasia was revealed on MR parotid sialography, and multiple hypoechoic areas were present in the bilateral parotid and submandibular glands on subsequent ultrasonography (US). These findings were consistent with those reported as characteristic of adult SS [5–7]. Therefore, juvenile SS was strongly suspected.

Ranulae are rare mucoceles caused by extravasation of saliva as a result of damage to the sublingual glands and/or obstruction of their ducts [9]. The etiology of ranula remains unknown. Ranulae have been described in association with trauma, anatomical variations, and chronic disease of the sublingual glands [8]. There are few reports on the relationship between SS and ranula [2, 9, 10]. However, in SS, lymphocytic infiltration can damage the ducts, inducing extravasation of saliva and thus mucus accumulation [9]. Therefore, sublingual gland damage resulting from SS could cause a ranula.

Thus, hypothesizing that SS might cause ranula development, we retrospectively investigated cases of patients with ranula who underwent MRI at our hospital. We present the details of the case of juvenile pSS that triggered this study and the results obtained in the subsequent retrospective study.

## Case series

### Case of juvenile pSS with plunging ranula (Figs. 1, 2; Case 1 in Table 1)

**Fig. 1:**
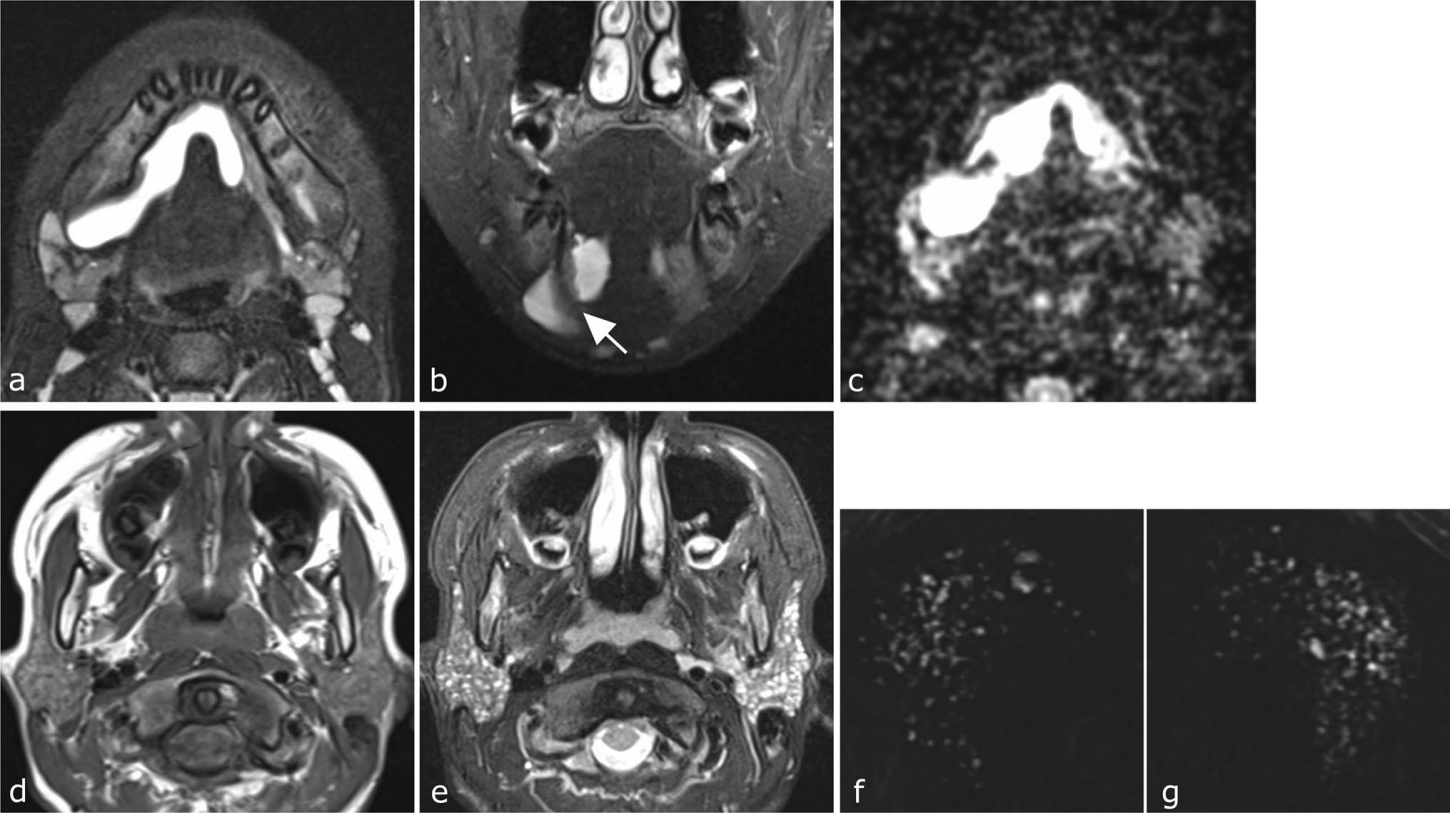
Magnetic resonance images of a 12 year-old boy with plunging ranula who was diagnosed with juvenile pSS (Case 1 in Table 1). **a**, **b** Axial (**a**) and coronal (**b**) fat-suppressed T2-weighted MR images show a homogeneous high-intensity, irregularly shaped, well-circumscribed cystic lesion extending from the bilateral sublingual space to the right submandibular space beyond the mylohyoid muscle (arrow). **c** Axial apparent diffusion coefficient (ADC) map shows a cystic lesion with extremely high ADC level. **d** Axial T1-weighted image shows slightly inhomogeneous parenchyma of the bilateral parotid glands. **e** Axial fat-suppressed T2-weighted image shows multiple dispersed high-intensity spots in parenchyma of bilateral parotid glands. **f**, **g** MR sialography shows punctate sialoectasia in the right (**f**) and left (**g**) parotid glands

**Fig. 2:**
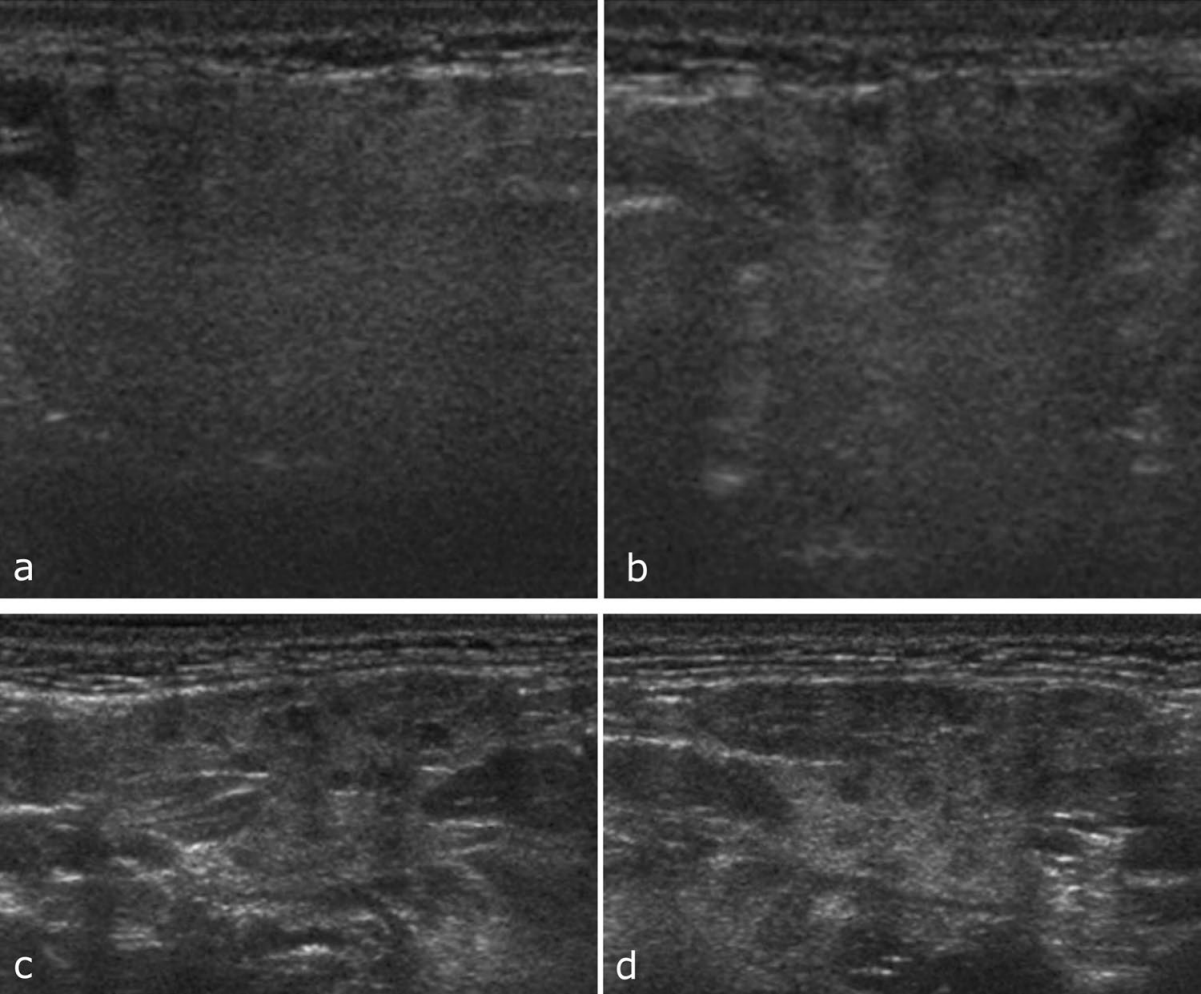
Ultrasonography of the patient shown in Fig. 1 (case 1 in Table 1). **a**, **b**, **c**, **d** Ultrasound images show multiple oval and polygonal hypoechoic areas in right (**a**) and left (**b**) parotid gland parenchyma and in right (**c**) and left (**d**) submandibular gland parenchyma

**Table 1:** Summary of ranula patients suspected of having Sjögren’s syndrome on the basis of MRI findings

Case	Age (years)	Sex	Type of ranula	Fat degeneration^a^ PG/SMG	Multiple High-intensity spots^b^ PG/SMG	Sialoectasia^c^ PG	Diagnosis of SS^d^
1	12	M	Plunging	−/−	+/−	Punctate	Definite SS
2	25	F	Simple	−/−	+/−	Punctate	Not exam
3	31	F	Simple	−/−	+/−	Punctate	Not exam
4	33	F	Simple	Mild/moderate	+/−	Punctate	Definite SS
5	38	F	Simple	Mild/severe	+/−	Globular	Not exam
6	41	F	Simple	Mild/mild	+/−	Not exam^e^	Definite SS
7	43	F	Simple	Moderate/mild	+/−	Globular	Definite SS
8	46	F	Simple	−/−	+/−	Punctate	Definite SS
9	48	F	Simple	Moderate/mild	−/−	Cavitary	Definite SS
10	51	F	Simple	Mild/mild	+/−	Punctate	Definite SS
11	65	F	Simple	−/−	+/−	Punctate	Not exam

^a^Classified into three grades (mild, moderate, severe) by two radiologists (Y.T. and M.S.) according to the degree of fat degeneration on T1-weighted and fat-suppressed T2-weighted images [5]. ^b^Evaluated by two radiologists (Y.T. and M.S.) on fat-suppressed T2-weighted images [5]. ^c^Sialoectasia was classified by two radiologists (Y.T. and M.S.) into three patterns (punctate, globular, cavitary) according to Rubin and Holt classification on the basis of X-ray sialography or MR sialography findings [5, 6]. ^d^Patients were clinically diagnosed by a rheumatologist as having SS according to the Japanese Ministry of Health criteria (1999) [25]. ^e^The patient underwent MR examination; however, MR sialography was not performed. PG, parotid gland; SMG, submandibular gland

A 12 year-old boy presented to our hospital with bilateral swelling of the oral floor that had been present for 4 years. A painless, soft, fluid-filled mass was observed on palpation. There were no clinical symptoms other than oral floor swelling. The patient had no history of recurrent parotitis, which is reported to be the most characteristic finding of juvenile pSS [1–4].

A ranula was suspected and MR examination was performed. Fat-suppressed T2-weighted images showed a homogeneous, high-intensity, irregularly shaped, well-circumscribed cystic lesion extending from the bilateral sublingual space to the right submandibular space beyond the mylohyoid muscle (Fig. 1a, b). The apparent diffusion coefficient of the cystic lesion on diffusion-weighted imaging was extremely high (Fig. 1c). Therefore, we diagnosed this cystic lesion as a plunging ranula. At this examination, T1-weighted images showed slightly inhomogeneous parenchyma of the bilateral parotid glands, and fat-suppressed T2-weighted images showed multiple dispersed high-intensity spots in the parenchyma of the bilateral parotid glands (Fig. 1d, e). MR sialography revealed punctate sialoectasia in the bilateral parotid glands (Fig. 1f, g). These findings were consistent with those reported as characteristic of SS [5, 6]. However, fat degeneration, which is also characteristic of the glands in SS, was not evident.

US revealed multiple oval and polygonal hypoechoic areas in the bilateral parotid and submandibular glands (Fig. 2a–d), as in adult SS [7].

These findings strongly suggested juvenile SS, and laboratory examinations were undertaken. Laboratory examinations were positive for antinuclear antibodies, SS-A, SS-B, and rheumatoid factor. Other notable findings included elevations of immunoglobulin G and amylase and the presence of anti-Smith antibodies. Saxon’s and Schirmer’s testing were negative. The patient had no sicca symptoms such as dry eyes or dry mouth. Biopsy of the labial glands was not performed because of lack of parental consent.

On the basis of imaging findings and laboratory data, a pediatric rheumatologist clinically diagnosed this patient with juvenile pSS.

The plunging ranula was treated with open fenestration. Concurrently, mizoribine and corticosteroid medications were initiated to treat SS. After treatment, the patient was asymptomatic. However, he developed parotitis for the first time about 9 months after the first medical examination.

### Case series study of SS with ranula (Table 1)

Hypothesizing that SS might cause ranula development, we retrospectively examined cases of ranula in patients who underwent MRI at our hospital between June 2008 and June 2020. We found that 11 of the 51 patients (> 20%) had findings suggestive of SS in the bilateral parotid and/or submandibular glands (Table 1). One of the 11 patients was the case of juvenile SS described above (Figs. 1, 2; Case 1 in Table 1); the other patients had adult SS (Cases 2–11 in Table 1).

**Fig. 3:**
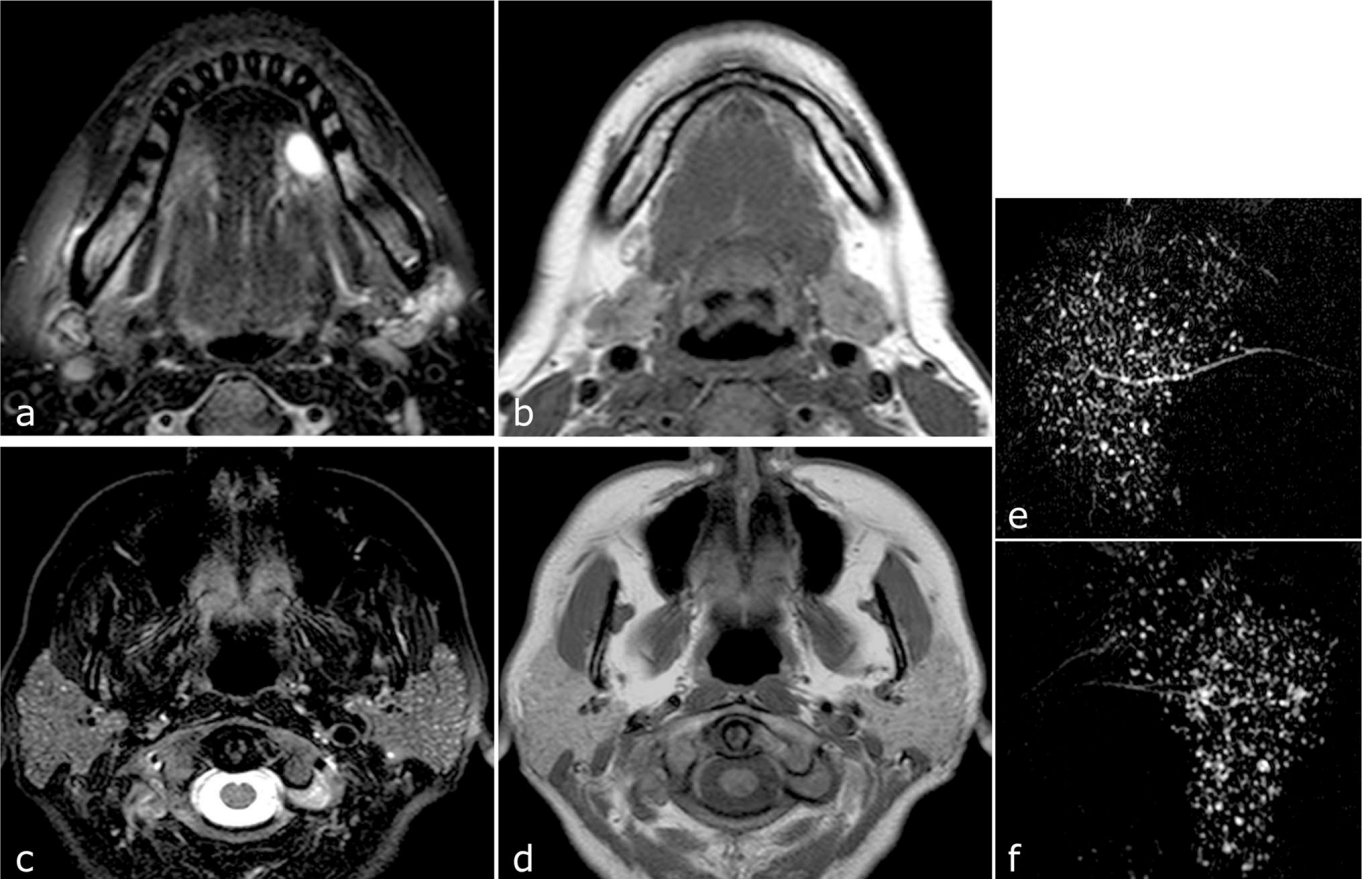
Magnetic resonance images of a 46 year-old woman with simple ranula who was diagnosed with pSS (Case 8 in Table 1). **a** Axial fat-suppressed T2-weighted MR images show a homogeneous, high-intensity, well-circumscribed cystic lesion in the left sublingual space. **b** Axial T1-weighted image shows relatively homogeneous parenchyma without fat degeneration of the bilateral submandibular glands. **c** Axial fat-suppressed T2-weighted image shows multiple very small high-intensity spots in parenchyma of the bilateral parotid glands. **d** Axial T1-weighted image shows relatively homogeneous parenchyma without fat degeneration of the bilateral parotid glands. **e**, **f** MR sialography shows punctate sialoectasia in the right (**e**) and left (**f**) parotid glands

**Fig. 4:**
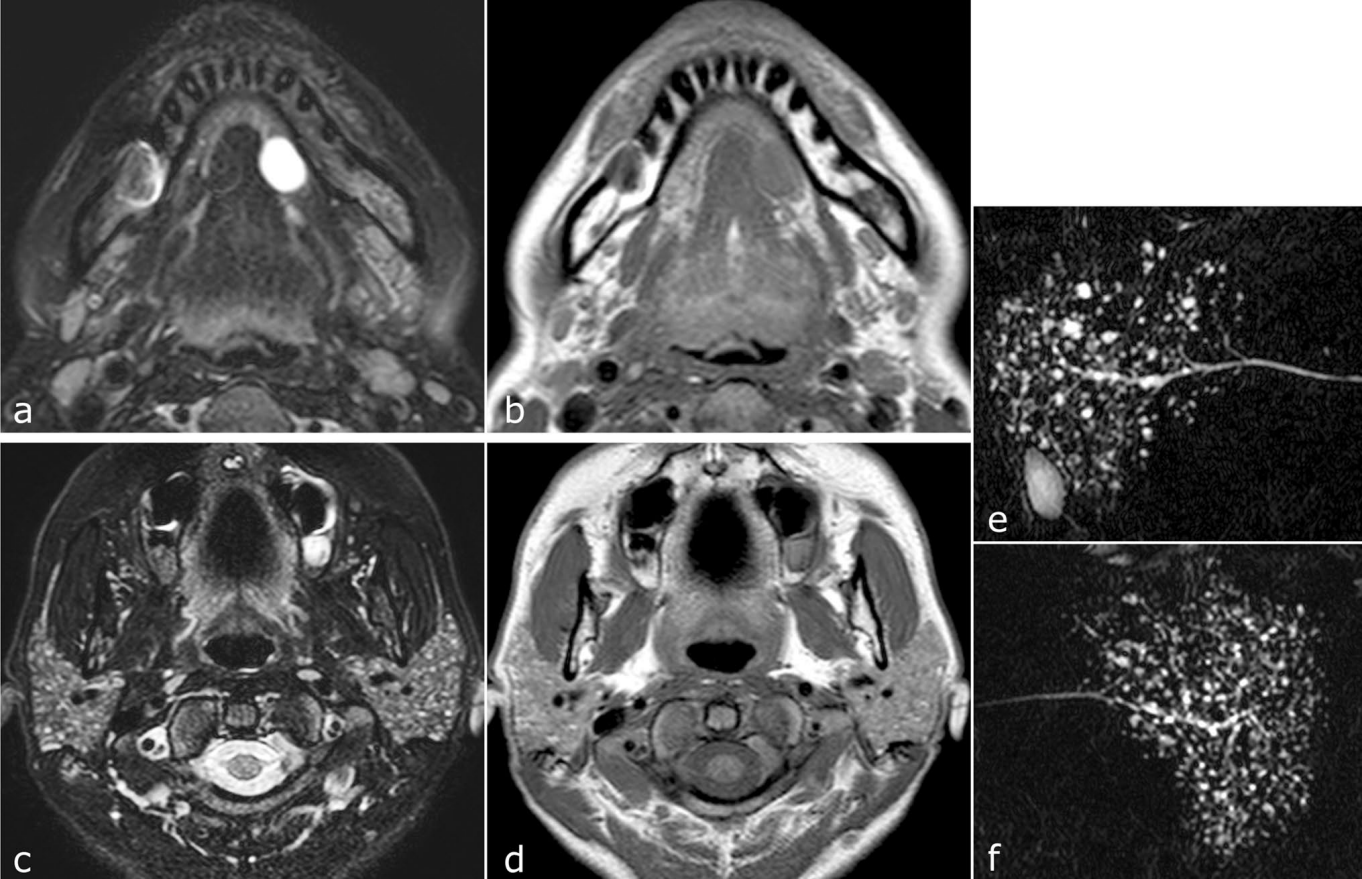
Magnetic resonance images of a 38 year-old woman with simple ranula who was not definitively diagnosed with SS but who had MRI findings strongly suggestive of SS (Case 5 in Table 1). **a** Axial fat-suppressed T2-weighted MR images show a homogeneous high-intensity, well-circumscribed cystic lesion in the left sublingual space. **b** Axial T1-weighted image shows bilateral atrophy of the submandibular glands resulting from severe fat degeneration. **c** Axial fat-suppressed T2-weighted image shows multiple very small high-intensity spots in parenchyma of the bilateral parotid glands. **d** Axial T1-weighted image shows slightly inhomogeneous parenchyma with fat deposition in the bilateral parotid glands **e**, **f** MR sialography shows punctate sialoectasia in right (**e**) and left (**f**) parotid glands.

Seven of the 11 patients were definitively diagnosed with SS by rheumatologists. In five of these seven patients, the MR examination for ranula led to the detection of SS (Fig. 3, Case 8 in Table 1). Four of the 11 patients were not definitively diagnosed with SS because they refused the necessary examinations. However, these patients had MRI findings strongly suggestive of SS (Fig. 4, Case 5 in Table 1).

## Discussion

Juvenile pSS is rare and its clinical symptoms and features are not well understood and are poorly defined [11–14]. In general, sicca symptoms are rarely observed in juvenile pSS patients. One reason for this absence may be that children are unable to accurately describe their symptoms [2]. Additionally, xerostomia appears after salivary gland dysfunction has progressed to some extent. Therefore, it is unlikely that xerostomia will be seen in children, whose dysfunction is generally not advanced. Thus, the clinical symptoms differ between juvenile and adult SS [1, 11, 13].

Multicenter surveys and literature reviews have reported that the most common clinical symptom in juvenile pSS is recurrent parotitis [1–4]. However, recurrent parotitis is a common disorder in childhood, caused most often by viral or bacterial infections [11, 15]. In juvenile pSS, other common conditions have been reported, such as fever of unknown origin, erythema, joint pain, fatigue, and multiple dental caries [1–4, 12–16]. However, standardized specific diagnostic criteria have not been established for juvenile pSS [4, 13, 15, 17]. The lack of specific diagnostic criteria in children makes juvenile pSS poorly known and probably underdiagnosed [4, 11, 13, 14]. The early diagnosis of juvenile pSS on the basis of clinical manifestations is challenging.

Biopsy of the labial glands is important in the diagnostic evaluation of SS patients [18]. However, this invasive examination is difficult to perform in all juvenile SS patients. Therefore, it is necessary to establish more sensitive child-specific diagnostic criteria for the unique patterns of early stages of the disease.

We propose the utility of noninvasive imaging examinations such as MRI and US. Although MRI and US findings are not currently included in the diagnostic criteria for SS, these imaging modalities have an important role in the evaluation of salivary glands and improve the diagnostic performance in adult SS [5–7, 19–21]. The usefulness of these imaging examinations in juvenile pSS is demonstrated in this study. Therefore, these imaging examinations are expected to become a useful noninvasive substitute for labial gland biopsy in both juvenile and adult SS. In addition, because these imaging modalities are noninvasive, they can be performed repeatedly. SS is an insidious and chronic disease; therefore, it is important to diagnose patients in the early stages and to follow them for a long time [16]. Follow-up imaging examinations are useful to assess the degree of progression in every salivary gland (parotid, submandibular, and sublingual glands).

Kimura et al. reported that fat degeneration of the glands is a characteristic finding on MR images in juvenile SS [22]. However, there were no findings suggestive of fat degeneration in this case. Fat degeneration is a conspicuous finding with progression of SS [5]. Because most juvenile SS patients are considered to be in the early stage of SS, it is conceivable that they are less likely to have associated fat degeneration. Fat degeneration is not always seen in the early stage of adult SS. Instead, multiple dispersed high-intensity spots in the parenchyma of the bilateral parotid glands are often observed on fat-suppressed T2-weighted MR images [5, 6], as in this case (Fig. 1e). The high-intensity spots on fat-suppressed T2-weighted MR images almost coincided with sialoectasia on MR sialography of the bilateral parotid glands [5, 6] (Fig. 1 e, f, g). Therefore, in juvenile SS, which is generally considered to be an early stage of SS, we think that multiple dispersed high-intensity spots in the bilateral parotid gland parenchyma on fat-suppressed T2-weighted MR images and/or sialoectatic change in the bilateral parotid glands on MR sialography are more characteristic findings than fat degeneration. However, further investigation with increased numbers of juvenile SS cases is needed to confirm this hypothesis.

Multiple dispersed high-intensity spots on fat-suppressed T2-weighted MR images and/or sialoectasia on MR sialography in the parotid gland parenchyma are also found in juvenile recurrent parotitis. However, the findings are unilateral in most cases of recurrent parotitis, whereas they are bilateral in SS, a difference that is useful in discriminating between the two diseases [22]. In this case, the findings were present in the bilateral parotid glands and there were no clinical signs of suspected parotitis. Therefore, parotitis was excluded and SS was suspected.

Additional US examinations showed findings suggestive of SS not only in the bilateral parotid glands but also in the bilateral submandibular glands (Fig. 2a-d). However, MRI did not reveal any findings of SS in the submandibular glands. US is a quick and inexpensive procedure compared with MRI and does not require patient sedation. Therefore, we think US is more appropriate than MRI for diagnosing juvenile SS, unless diagnosis of a ranula is necessary [11, 14].

Ranulae are clinically subdivided into simple (intraoral) and plunging (cervical) according to the extent of the pseudocyst. The simple ranula is present within the sublingual space, whereas the plunging ranula extends beyond the mylohyoid muscle to the submandibular space and adjacent structures in the neck, as in our present case (Fig. 1b) [8, 23, 24].

Ranulae most frequently occur in patients under the age of 30 years and rarely occur in young children [8]. However, Than et al. reported many pediatric ranula cases. According to that report, simple ranulae are more common and occur at a younger average age than plunging ranulae [24].

Ranulae are caused by extravasation of saliva resulting from damage to the sublingual glands and/or obstruction of their ducts. The pathological feature of SS is periductal lymphocytic infiltration; the resulting damage to the duct is presumed to induce the extravasation of saliva and thus mucus accumulation [9]. Therefore, the ranula is likely to be an important clinical sign of SS. Although there are few reports on the relationship between SS and ranula [2, 9, 10], Sato et al. reported that extravasation of saliva from fragile ducts often occurs only in the early stages of SS [9]. Ranulae are less likely to develop in advanced stages because there is insufficient saliva to form a ranula when there is acinar atrophy and loss resulting from chronic lymphocyte aggregation [9]. Therefore, a ranula is one of the characteristic symptoms of early SS.

Our case series study revealed that 11 of the 51 patients with ranula had findings suggestive of SS, indicating that SS may be accompanied by ranula in both adults and children. In addition, the age of ranula development in patients suspected of having SS was relatively young, and the SS stage of the patients who underwent MRI at our hospital was not advanced (Table 1). This finding suggests that ranulae occur in the early stages rather than in the advanced stages of SS, as Sato et al. stated [9].

In some cases, as in the juvenile pSS case presented here, a ranula may be the only clinical sign that leads to early detection of SS. Therefore, patients with ranulae, whether adults or children, should undergo careful assessment of not only the sublingual glands but also the parotid and submandibular glands with MRI and/or US to investigate possible SS.

In addition, we recommend the inclusion of imaging examinations in the diagnostic criteria for juvenile and adult SS to facilitate diagnosis at an earlier stage of the disease.

Currently, diagnosis of juvenile pSS is more difficult than that of juvenile sSS or adult SS. However, if imaging examinations such as MRI and/or US are used to diagnose SS, juvenile pSS can be diagnosed earlier. Earlier diagnosis will allow earlier initiation of treatment and prevention of progression in the exocrine glands and complications in other organs.
